# MR-guided renal denervation: first experience in pigs

**DOI:** 10.1186/1532-429X-16-S1-P33

**Published:** 2014-01-16

**Authors:** Florian Bönner, Nico Janzarik, Jouke Smink, Sascha Krüger, Christian Meyer, Dong-In Shin, Malte Kelm, Mirja Neizel-Wittke

**Affiliations:** 1 Department of Cardiology, Pneumology and Vascular Medicine, University Düsseldorf, Düsseldorf, Germany; 2 Philips Healthcare, Best, Netherlands

## Background

Renal nerve ablation under fluoroscopy has emerged as a promising therapy in patients non-responding to oral anti-hypertensive medications. However, a considerable drawback of this technique is the lack of exact and immediate therapy control. CMR has the advantage of simultaneously combining functional imaging, anatomic and intraprocedural guidance in one examination without radiation and ionidied contrast agents.

## Methods

3 pigs (Munich mini-pigs, 55-75 kg BW) were investigated under general anaesthesia and mechanical ventilation after canulation of the right femoral artery with a 9 french introducer sheath. Real-time experiments were performed on a 1.5 Tesla MRI-System (Achieva, Philips, Best, Netherlands) equipped with inroom monitors, non/invasive patient monitoring (ECG, blood pressure, oxymetry) and an interventional software platform (iSuite, Philips, Best, Netherlands). After assessing baseline images for navigation ("waypoints"), oedema imaging and non contrast angiograms of the renal artery, catheter guidance was coordinated between the interventional- and the MRI -operator based on a steady-state free precession real-time imaging sequence. Renal denervation (4-5 × 8 Watt for approximately 2 min) was performed using a human application approved 8 F MR conductable non-irrigated ablation catheter (IMRICOR, Burnsville, MN, USA). Thereafter, renal artery patency, flow and development of vessel wall oedema were assessed for prove of sufficient renal artery ablation and possible side effects. Finally, kidneys, renal arteries and surrounding tissue were explanted and assessed histologically.

## Results

Renal artery ablation was feasible using the MR conditional ablation catheter (IMRICOR, Burnsville, MN, USA) in all cases with survival of all animals. Non-contrast angiography of renal arteries displayed patency accompanied by equal flow conditions before and after the ablation in all cases. Oedema of the renal artery wall could be demonstrated after renal denervation. At pathology no signs of bleeding and perforation could be observed. At histology sufficient ablation lesions could be demonstrated.

## Conclusions

MR-guided renal denervation is feasible and effective. Handling of the MR-interventional system with iSuite is comfortable for both, the interventionalist and the MR-Operator, and therefore provides a low learning curve. A major advantage of MR-guided interventions is the ability to simultaneously monitor tissue alterations (Temperature, oedema etc.) with high resolution.

## Funding

None.

